# Care provided and care setting transitions in the last three months of life of cancer patients: a nationwide monitoring study in four European countries

**DOI:** 10.1186/1471-2407-14-960

**Published:** 2014-12-16

**Authors:** Winne Ko, Luc Deliens, Guido Miccinesi, Francesco Giusti, Sarah Moreels, Gé A Donker, Bregje Onwuteaka-Philipsen, Oscar Zurriaga, Aurora López-Maside, Lieve Van den Block, on behalf of EURO IMPACT

**Affiliations:** End-of-Life Care Research Group, Vrije Universiteit Brussel (VUB), Brussels and Ghent University, Ghent, Belgium; Department of Medical Oncology, University Hospital Ghent, Ghent, Belgium; EMGO Institute for Health and Care Research, Department of Public and Occupational Health, and Palliative Care Expertise Centre, VU University Medical Centre, Amsterdam, the Netherlands; Clinical and Descriptive Epidemiology Unit, Cancer Prevention and Research Institute, ISPO, Florence, Italy; Public Health and Surveillance, Scientific Institute of Public Health, Brussels, Belgium; NIVEL, Netherlands Institute for Health Services Research, Utrecht, the Netherlands; Health Department, Public Health Directorate General, Valencia, Spain; Spanish Consortium for Research in Epidemiology and Public Health, CIBERESP, Barcelona, Spain; Department of Family Medicine and Chronic Care, Vrije Universiteit Brussel (VUB), Brussels, Belgium

## Abstract

**Background:**

This is an international study across four European countries (Belgium[BE], the Netherlands[NL], Italy[IT] and Spain[ES]) between 2009 and 2011, describing and comparing care and care setting transitions provided in the last three months of life of cancer patients, using representative GP networks.

**Methods:**

General practitioners (GPs) of representative networks in each country reported weekly all non-sudden cancer deaths (+18y) within their practice. GPs reported medical end-of-life care, communication and circumstances of dying on a standardised questionnaire. Multivariate logistic regressions (BE as a reference category) were conducted to compare countries.

**Results:**

Of 2,037 identified patients from four countries, four out of five lived at home or with family in their last year of life. Over 50% of patients had at least one transition in care settings in the last three months of life; one third of patients in BE, IT and ES had a last week hospital admission and died there. In the last week of life, a treatment goal was adopted for 80-95% of those having palliation/comfort as their treatment goal. Cross-country differences in end-of-life care provision included GPs in NL being more involved in palliative care (67%) than in other countries (35%-49%) (OR 1.9) and end-of-life topics less often discussed in IT or ES. Preference for place of death was less often expressed in IT and ES (32-34%) than in BE and NL (49-74%). Of all patients, 88-98% were estimated to have distress from at least one physical symptom in the final week of life.

**Conclusion:**

Although palliative care was the main treatment goal for most cancer patients at the end of life in all four countries, frequent late hospital admissions and the symptom burden experienced in the last week of life indicates that further integration of palliative care into oncology care is required in many countries.

## Background

While survival rates for cancer have increased considerably, it is still one of the leading causes of death in many developed countries [1, 2]. For people suffering from an advanced form of cancer, palliative care is recognised as the preferred form of care at the end of life (EOL). The World Health Organisation (WHO) defines palliative care as ‘an approach that improves the quality of life of patients and their families facing the problems associated with life-threatening illness, through the prevention and relief of suffering by means of early identification and impeccable assessment and treatment of pain and other problems, physical, psychosocial and spiritual’ [3]. Within the framework of palliative care, several topics are considered important [4], such as the use of palliative care services, communication, advance care planning and the circumstances of dying. Palliative care has been developed differently in different countries in terms of processes, structures, policies and resources that support its delivery [5–7]. However, cross-country population-based studies aimed at describing these variations in actually delivered EOL care for people with cancer in Europe are scarce [4, 8, 9]. Existing studies are often restricted in the themes covered e.g. pain [10] or place of death [11] or in terms of the study population e.g. hospital or hospice settings [12, 13].

Comparative and nationwide EU studies measuring across different care settings and cancer types could inform us on organising palliative care for dying cancer patients. General practitioners (GPs) are highly accessible in Europe and they have a central coordinating role in patient care in most EU countries [14]. GPs can generally provide a good public health perspective on end-of-life care in their own country. In this study, we aim to use nationwide networks of GPs in four EU countries (Belgium [BE], the Netherlands [NL], Italy [IT] and Spain [ES]) to describe and compare the medical care process, patient-GP communication processes and the actual circumstances of dying of cancer patients in the four countries studied.

## Methods

### Design

Data were collected within the European Sentinel GP Networks Monitoring End-of-Life Care (EURO SENTI-MELC) study, which continuously monitored EOL care via the use of representative networks of GPs in 2009–2011 in four EU countries: 2009–2010 in Belgium, the Netherlands and Italy, and 2010–2011 in Spain. All countries were sampled nationwide except Spain, where two regions (North: Castilla y León and East: Valencia) were included.

Both the study protocol and an earlier wave of data (2008) comparing deceased cancer patients in Belgium and the Netherlands have been published [4, 15, 16]. Using a mortality follow-back questionnaire design, GPs reported all deaths in their practices (age ≥ 18 yrs.) on the EOL care provided in the final months of life on standardised forms.

### Palliative care in the four countries studied

Access to palliative care has been recognised as a right in all countries except NLalthough there it is covered by the national health insurance. In all four countries, some type of plan or national guideline for palliative care is available [17]; it is therefore understood that patients in these countries have access to palliative care. However, none of these countries recognise palliative care as a medical specialty and the model of palliative care provision varies. For example, GPs co-ordinate care in Italy and Spain in the primary care settings, while in Belgium the care is often shared in the context of a multidisciplinary team and in the Netherlands palliative care is provided mainly by the GPs in consultation with specialist teams [7, 17].

### Setting and participants

In general the GP networks are representative of all GPs in the country in terms of age, gender and geographical distribution. Sentinel surveillance systems of GPs are used to provide information regarding the whole patient population in a country, particularly in countries where general practice is highly accessible. Percentages of the general population covered by the GP sentinel networks are 1.75% of the total Belgian population, 0.8% of the Dutch population, 2.2% in Valencia, 3.5% of the adult population in Castilla y Leon and 4% of the Italian population (per health district). For the specific purpose of our study we additionally analysed the representativity of the networks to cover all deaths in the country. These results were published earlier [16] showing that data collected from the GP networks had significant but small differences from available mortality statistics or death certificate studies in terms of age, gender and place of death [16]. In all countries GPs can identify deaths due to cancer and non-cancer and those dying at home as well as in institutional settings. GPs appear to underreport a limited number of deaths ie non-sudden hospital deaths and deaths of people under 65 years in Belgium, and possibly also sudden hospital deaths in all countries [16]. Apart from in Italy, the networks in all countries are existing Sentinel GP networks involved in the surveillance of different health related topics [16]. In 2009, the number of GPs participating in the study were 199 (1.8%) in Belgium, 59 (0.8%) in the Netherlands and 149 (4.3%) in Italy. In 2010, the figures were 189 (1.5%) in Belgium, 63 (0.8%) in the Netherlands, 94 (2.7%) in Italy, and 173 in Spain (114 (3.4%) in Castilla and León, 59 (3%) in Valencia).

For this study, we included cancer patients only i.e. cancer as GP-reported ‘underlying cause of death’, and excluded those judged to have died ‘suddenly and totally unexpectedly’ by the GP. Nursing home deaths from the Netherlands were excluded since GPs discontinued their care after the transition to a nursing home where care is taken over by elderly care physicians.

### Data collection and measurements

To minimise recall bias, physicians recorded deaths immediately after the patient died. Paper-based forms were administered in Belgium, the Netherlands and Castilla y León, whereas a web-based registration was adopted in Italy and an electronic registry in Valencia. To ensure the quality of data collected, instructions on filling out the form were sent to GPs at the beginning of the year in all countries. Every GP is asked to fill in a weekly, standardised registration form, whether or not there was a deceased patient. Where a patient had died during that week, the GP filled in the questions concerning care and dying. Only if GPs registered weekly for 26 weeks or more were their data included in the databases.

GPs answered questions about the final three months of life of deceased patients. These questions were derived from and developed in previous research [4, 16, 18–20]. A validated item, the MSAS-GDI, was included in the questionnaire [21]. Other items in the questionnaire had been pre-tested in the pilot studies with experts to increase validity and reliability. Further details can be found in the methodological paper published earlier [16].

Questions were classified into three palliative care domains measuring:

- **Medical care processes (last three months and last week of life):** number of GP-patient contacts, transitions between care settings, use of specialist palliative care services, use of GP palliative care, costs and burden of informal caregivers as judged by the GP, treatment goals (cure, life-prolonging, palliative care in the final three months of life), terminal hospital admission (i.e. dying in the hospital) and timing of this admission
- Communication processes:

1. Patient-GP conversations about primary diagnosis, incurability of illness, life expectation, possible medical complications, physical complaints, psychological problems, social problems, spiritual problems, options for palliative care, burden of treatments (options: *yes, no, not applicable*)
2. EOL preferences for place of death and medical treatment as known to the GP.

- Circumstances of dying in the last week of life

Physical and psychological symptom distress was measured using the Memorial Symptom Assessment Scale- Global Distress Index (MSAS-GDI) [21]. GPs estimated:

1. Physical symptom distress: lack of appetite, lack of energy, pain, drowsiness, constipation, dry mouth, difficulty in breathing (dyspnoea) (GPs first indicated the presence of symptoms as *yes, no or unknown*; if yes, they indicated *not at all, a little bit, somewhat, quite a bit or very much*)
2. Psychological symptom distress: sadness, worry, irritation, nervousness (GPs first indicated the presence of symptoms as *yes, no or unknown*; if yes, they indicated *rarely occasionally, frequently, or almost constantly*).

Basic information about the patient (age, gender, type of malignancy, longest place of residence in the last year and place of death) was also registered by the GPs. Questions were first developed in Dutch and subsequently translated into French and English, and from English to Italian and Spanish through forward-backward procedures [16].

### Ethical approval

In Belgium the protocol of the study was approved by the Ethical Review Board of Brussels University Hospital of the Vrije Universiteit Brussel (2004). In Italy, ethics approval for data collection was obtained from the Local Ethical Committee ‘Comitato Etico della Azienda U.S.L. n. 9 di Grosseto’, Tuscany (2008). Ethical approval was not required for posthumous collection of anonymous patient data in the Netherlands [22, 23] or Spain [24–26]. Patients and GPs remain anonymous to researchers and the institutes.

### Statistical analysis

Descriptive statistics were employed to show the distribution of characteristics of the study population and Pearson’s chi-squared tests to detect cross-country differences (p < .001).

Further multivariate logistic regression analyses (with Belgium as the reference category) were performed to study the cross-country variations in EOL care controlling for differences in place of death, age and sex and, for the analyses regarding circumstances of dying, we additionally controlled for the number of GP contacts in the last week of life. Odds ratios (ORs) and 95% confidence interval (95% C.I.) were calculated. All analyses were completed with SPSS20.0 (IBM Corp, Armonk, NY)

## Results

### Characteristics of patients

A total of 2,037 deceased cancer patients were identified from four countries (Table 1). Mean age was 73.1 years. Over 85% of cancer patients lived at home or with family in their last year of life. In Belgium and the Netherlands, 11% and 9% lived in a care home whereas the figure was 3% in Italy and 4% in Spain.

**Table 1:** **Characteristics of the study population: non-sudden cancer deaths (** ***N*** **= 2037)**

Characteristics	Belgium (***N*** = 595)	The Netherlands (***N*** = 335)	Italy (***N*** = 830)	Spain (***N*** = 277)	***p***-value ^b^
	***N***(%)	***N***(%)	***N***(%)	***N***(%)	
**Age group**					.156
18-64	160(27.1)	90(26.9)	195(23.5)	59(21.3)	
65-74	131(22.2)	94(28.1)	210(25.3)	65(23.5)	
75-84	184(31.2)	103(30.7)	257(31.0)	97(35.0)	
85 or above	115(19.5)	48(14.3)	168(20.2)	56(20.2)	
Mean	72.5	71.9	73.6	74.2	
**Gender**					<.001
M	328(55.1)	177(53.2)	448(54.0)	186(67.6)	
F	267(44.9)	156(46.8)	382(46.0)	89(32.4)	
**Types of malignancy**					<.001
Lung	148(24.9)	80(25.0)	182(26.5)	53(19.8)	
Breast	51(8.6)	33(10.3)	57(8.3)	11(4.1)	
Colorectal	69(11.6)	38(11.9)	102(14.8)	49(18.3)	
Prostate	25(4.2)	23(7.2)	35(5.1)	29(10.8)	
Other	302(50.8)	146(45.6)	312(45.3)	126(47.0)	
**Longest place of residence in the last year**				<.001	
Home or with family	516(87.2)	300(90.6)	799(96.5)	258(95.2)	
Care home	65(11.0)	30(9.1)	21(2.5)	10(3.7)	
Other	11(1.9)	1(0.3)	8(1.0)	3(1.1)	
**Place of death**					<.001
Home	196(33.2)	194(57.9)	377(45.5)	139(51.1)	
Nursinghome/Residential home for older people	71(12.0)	28(8.4)	41(5.0)	11(4.0)	
Hospital	198(33.5)	58(17.3)	312(37.7)	95(34.9)	
Palliative Care Unit/Hospice	122(20.6)	54(16.1)	94(11.4)	26(9.6)	
Elsewhere	4(0.7)	1(0.3)	4(0.5)	1(0.4)	

^a^Missing for age group: n = 5, gender: n = 4, Type of malignancy: n = 166, Longest place of residence last year: n = 15, Place of death: n = 11.

^b^χ^2^test on cross-country differences.

Home deaths were more common in the Netherlands (57.9%) and Spain (51.1%).Except for the Netherlands (17%), more than one third of cancer patients died in hospital in all countries (34%[BE], 38%[IT] and 35%[ES]).

### Medical care processes at the end of life

During the last three months of life, GPs had more than three contacts with patients in 23% (BE), 35% (ES), 42% (NL) and 58% (IT) of cases (Table 2). In all countries, more than half of cancer patients (between 52.6% and 69%) had at least one transition between care settings in their last three months of life. Specialist palliative care services were used in 37% of cases in NL, compared with 58%, 62% and 65% in ES, IT and BE respectively. GP palliative care was provided until death in 67% of cases in NL, compared with 49%, 44% and 35% in ES, BE and IT. Five percent of patients in Spain had difficulty in covering costs, differing from 20%, 38% and 43% in BE, NL and IT. From 31% in NL to 35% (BE), 42% (ES) and 78% (IT) of informal caregivers in the four countries were perceived to be overburdened.

**Table 2:** **Characteristics of the medical care processes at the end of life**

	Belgium (***N*** = 595)	The Netherlands (***N*** = 335)		Italy (***N*** = 830)		Spain (***N*** = 277)		***p***-value ^a^
Characteristics	***N***(%)	***N***(%)	OR ^b^ (95% C.I.)	***N***(%)	OR ^b^ (95% C.I.)	***N***(%)	OR ^b^ (95% C.I.)	
**During the last three months of life:**								
More than three GP-patient contacts^d^	137(23.0)	140(41.8)	**2.2(1.6-3.0)**	480(57.8)	**4.6(3.6-5.9)**	98(35.4)	**1.7(1.3-2.4)**	<.001
At least one transition to another care setting	407(69.0)	173(52.6)	0.8(0.6-1.2)	534(64.4)	0.9(0.7-1.3)	156(57.4)	0.7(0.5-1.1)	<.001
Specialist palliative care services initiated	370(65.1)	119(37.0)	**0.2(0.2-0.3)**	502(62.7)	1.2(0.9-1.6)	160(57.8)	1.0(0.7-1.4)	<.001
GP provided palliative care until death	262(44.0)	219(67.0)	**1.9(1.3-2.7)**	290(35.0)	**0.4(0.3-0.6)**	129(49.4)	0.8(0.5-1.2)	<.001
Difficult in covering care costs	92(20.3)	107(38.4)	**2.5(1.8-3.6)**	306(42.5)	**3.0(2.3-4.1)**	11(4.9)	**0.2(0.1-0.4)**	<.001
Informal caregivers feeling overburdened	183(34.9)	92(30.9)	0.7(0.5-1.00)	590(77.9)	**6.1(4.7-8.0)**	99(41.8)	1.1(0.8-1.6)	<.001
Palliation as important treatment goal (*v* curative/prolonging life)	305(58.1)	223(76.4)	**2.2(1.6-3.1)**	418(60.9)	1.2(0.9-1.5)	138(63.3)	1.2(0.9-1.8)	<.001
**During the last week of life:**								
One or more GP-patient contacts	448(75.3)	286(85.4)	1.3(0.9-1.9)	625(75.3)	0.8(0.6-1.1)	178(64.3)	**0.4(0.3-0.6)**	<.001
At least one transition to another care setting	145(24.7)	56(16.8)	0.9(0.6-1.3)	159(19.5)	0.8(0.6-1.1)	71(27.4)	**1.5(1.1-2.3)**	0.002
Terminal hospital admission	198(33.5)	58(17.3)	**0.4(0.3-0.6)**	312(37.7)	1.2(1.0-1.5)^c^	95(34.9)	1.1(0.8-1.6)	<.001
Admission during last week (*v*before last week)^e^	70(35.7)	22(38.6)	1.2(0.6-2.2)	100(33.2)	1.0(0.6-1.4)	44(47.8)	**1.8(1.1-3.1)**	.084
Palliation as important treatment goal (*v* curative/prolonging life)	522(91.9)	296(94.9)	1.4(0.8-2.6)	487(80.0)	**0.4(0.3-0.6)**	206(89.6)	0.9(0.5-1.5)	<.001

*Abbreviations: GP* general practitioner, *OR* odds ratio.

Missing data:

During last 3 months of life: more than three GP-patient contacts: n = 1%; at least one transition: n < 1%; specialist palliative care initiated: n < 4%; GPs’ provision of pall care until death: n < 3%; difficult in covering care costs: n < 18%; informal caregivers feeling overburdened: n < 11%.

During last week of life: transferred at least once: n < 3%; terminal hospital admission: n < 1%; admission during last week:n < 68%, palliation as important treatment goal during last week: n < 16%.

^a^χ^2^test on cross-country differences.

^b^Odd Ratios from multivariate logistic regression models. For these analyses, we compared end-of-life care between patients with cancer in the four countries, with Belgium as the reference category, and adjusted differences in place of death, age, gender and the types of malignancy. Odds ratios in bold are statistically significant at p < 0.05.

^c^Place of death not controlled for in multivariate analyses.

^d^median number of contacts during the last three months of lifeacross countries was 3.

^e^For patients who died in hospitals.

In the last week of life, GPs had more than one contact with patients in two thirds of cases in all countries and 17% [NL] to 27% [ES] of patients were transferred to another setting. Terminal hospital admission was experienced by one in three patients in BE, IT and ES respectively, and by 17% in NL. These admissions occurred in the last week of life in one out of three cases except in ES where it was 48%. For all countries, palliative care was the main treatment aim for most patients in the last week of life (about 90% of patients in BE, NL and ES, and 80% in IT).

After controlling for differences in patient characteristics, variations in GP contacts in the last three months of life remained significant, as did the use of GP and specialist palliative care services.

### Communication processes at the end of life

In all countries, a large majority of GPs had discussed one or more topics (between 89% [IT] and 98% [NL]) (Table 3). Most GPs in NL (95%) discussed primary diagnosis with patients, compared with84%, 71% and 66% respectively in BE, ES and IT. Physical complaints were also frequently discussed (between 83% [IT] and 96% [NL]). Over half of patients had conversations with GPs on psychological problems (between 60% [IT] and 87% [NL]). One out of three patients talked about social problems with their GPs in IT (35%) and ES (34%), compared with 57% and 70% in BE and NL respectively. ‘Spiritual problems’was the topic least often discussed in all countries, from about 15% in IT and ES to 32% in BE and slightly over half (54%) in NL. Except in IT (37%), over two-thirds of GPs in all countries had conversations on the options of palliative care (from 67% [ES] to 70%[BE] and 88% [NL]).

**Table 3:** **Characteristics of communication processes at the end of life**

	Belgium (***N*** = 595)	The Netherlands (***N*** = 335)		Italy (***N*** = 830)		Spain (***N*** = 277)		***p***-value ^a^
Characteristics	***N***(%)	***N***(%)	OR (95% C.I.)	***N***(%)	OR ^b^ (95% C.I.)	***N***(%)	OR ^b^ (95% C.I.)	
**GP-patient conversations about:**								
Primary diagnosis	474(84.2)	303(95.0)	**3.1(1.8-5.4)**	505(66.4)	**0.3(0.3-0.5)**	157(70.7)	**0.4(0.3-0.6)**	<.001
Incurability of illness	416(74.4)	298(94.6)	**5.0(2.9-8.6)**	345(46.2)	**0.3(0.2-0.4)**	95(45.0)	**0.3(0.2-0.4)**	<.001
Life expectation	363(64.5)	282(89.5)	**4.1(2.7-6.2)**	277(37.0)	**0.3(0.2-0.4)**	56(27.1)	**0.2(0.1-0.3)**	<.001
Possible medical complications	393(70.1)	267(86.4)	**2.6(1.8-3.9)**	441(58.7)	**0.6(0.4-0.7)**	137(62.8)	**0.7(0.5-0.95)**	<.001
Physical complaints	514(90.7)	306(95.9)	**2.3(1.2-4.4)**	632(83.0)	**0.5(0.4-0.7)**	208(90.4)	0.9(0.5-1.6)	<.001
Psychological problems	416(74.3)	272(86.9)	**2.1(1.4-3.1)**	442(59.1)	**0.5(0.4-0.7)**	146(66.2)	**0.7(0.5-0.9)**	<.001
Social problems	284(56.5)	202(70.1)	**1.8(1.3-2.6)**	249(34.5)	**0.4(0.3-0.5)**	63(34.4)	**0.4(0.3-0.6)**	<.001
Spiritual problems	169(32.4)	156(54.4)	**2.1(1.5-2.9)**	104(14.4)	**0.3(0.3-0.5)**	27(14.7)	**0.3(0.2-0.5)**	<.001
Options for palliative care	389(70.0)	272(88.0)	**2.7(1.8-4.0)**	267(36.4)	**0.2(0.2-0.3)**	138(66.7)	0.8(0.5-1.1)	<.001
Burden of treatments	397(72.6)	244(81.6)	**1.6(1.1-2.3)**	367(50.0)	**0.4(0.3-0.5)**	136(68.3)	0.8(0.5-1.1)	<.001
**Overall**: One or more of these topics was discussed	447(94.1)	259(97.7)	2.2(0.9-5.4)	586(88.7)	**0.5(0.3-0.7)**	133(95.7)	1.2(0.5-2.9)	<.001
**End-of-lifepreferences----Patient everexpressed a preference:**								
For place of death	293(49.3)	245(73.8)	**2.3(1.7-3.1)**	267(32.2)	**0.4(0.3-0.5)**	85(34.0)	**0.4(0.3-0.6)**	<.001
About a medical treatment	225(40.5)	198(65.1)	**2.4(1.7-3.2)**	118(18.2)	**0.3(0.3-0.4)**	29(14.3)	**0.2(0.1-0.4)**	<.001

Missing data:

Prior to last month: Diagnosis, possible medical complication, psychosocial problems: n < 10%; incurability of illness, life expectancy: n < 11%; physical problems: n < 8%, social problems; n < 17%; spiritual problems, n < 16%; options of palliative care, n < 12%; Burden of treatment: n < 13%; one or more issues discussed: n < 23%.

Preference for place of death: n < 13%; preference about medical treatment: n < 17%.

^a^χ^2^test on cross-country differences.

^b^Odds ratios from multivariate logistic regression models. For these analyses, we compared end-of-life care between patients with cancer in the four countries, with Belgium as the reference category, and adjusted differences in place of death, age, gender and the types of malignancy. Odds ratios in bold are statistically significant at p < 0.05.

Other than in NL (74%), fewer than half of cancer patients expressed a preference for place of death (between 32% [IT] and 49% [BE]). Fewer than one-fifth of patients in ES (14%) and IT (18%) indicated at any time a preference about medical treatment, whereas the figures were 41% and 65% in BE and NL.

When other factors were controlled for, seven out of 10 of the aforementioned differences remained significant and topics such as the incurability of illness (more in NL, 5.0; less in IT &ES, 0.3) and options for palliative care (more in NL, 1.6; less in IT, 0.4) were less often discussed in IT and ES than in BE and the NL. The higher frequencies of discussions in NL on preference for place of death (OR 2.3, less in IT & ES, 0.4) and medical treatment (OR 2.4, less in IT 0.3 and ES 0.2) remained.

### Circumstances of dying in the last week of life (Table 4)

Suffering from physical symptoms was common among cancer patients; from 88% (IT) to 92% (BE & NL) and 99% (ES) experienced at least one symptom. Over 70% of the patients in all countries experienced lack of energy, except in ES (57%). From 64%, 87% to 100% of patients in respectively in BE, IT and ES were judged to be distressed by at least one psychological symptom, and the figure was 48% for NL. Respectively 66% [IT], 75% [BE], 79% [SP] and 87% [NL] of patients in all countries died in their preferred place if their wishes were known to the GP.

**Table 4:** **Circumstances of the dying process**

	Belgium (***N =*** 595)	The Netherlands (***N*** = 335)		Italy (***N*** = 830)		Spain (***N*** = 277)		***p***-value ^a^
Variable	***N***(%)	***N***(%)	OR ^b^ (95% C.I.)	***N***(%)	OR ^b^ (95% C.I.)	***N***(%)	OR ^b^ (95% C.I.)	
**Physical symptom distress in last week of life**								
GP could make estimation^c^	520(87.4)	285(85.1)	**0.2(0.1-0.3)**	702(84.6)	**0.5(0.4-0.8)**	231(83.4)	0.8(0.5-1.3)	.355
Distress from at least one physical symptom	412(91.6)	215(91.9)	1.1(0.6-1.9)	571(88.4)	0.7(0.4-1.1)	134(98.5)	**5.8(1.4-24.8)**	.002
Lack of appetite	280(60.1)	120(49.6)	0.7(0.5-1.0)	299(47.8)	**0.6(0.4-0.8)**	67(49.6)	0.7(0.4-0.992)	.001
Lack of energy	344(72.0)	186(73.5)	1.1(0.8-1.7)	482(73.8)	1.0(0.8-1.3)	83(56.8)	**0.5(0.3-0.8)**	.001
Pain	93(23.7)	56(26.8)	1.4(0.9-2.2)	236(38.2)	**2.0(1.5-2.8)**	46(48.4)	**3.2(2.0-5.2)**	<.001
Feeling drowsy	142(32.9)	60(29.0)	1.0(0.7-1.5)	182(29.4)	0.9(0.7-1.2)	18(25.0)	0.6(0.3-1.1)	.438
Constipation	57(17.8)	22(13.8)	0.7(0.4-1.3)	158(27.7)	**1.7(1.2-2.4)**	26(32.1)	**2.1(1.2-3.8)**	<.001
Dry mouth	73(20.4)	44(22.6)	1.1(0.7-1.7)	177(30.9)	**1.6(1.1-2.2)**	34(39.5)	**2.3(1.3-3.8)**	<.001
Difficulty breathing	140(36.2)	58(32.8)	1.1(0.7-1.6)	243(40.6)	1.3(1.0-1.8)	40(60.6)	**2.5(1.4-4.4)**	<.001
**Psychological symptom distress in last week of life**								
GP could make estimation^c^	487(81.8)	258(77.0)	**0.2(0.1-0.3)**	649(78.2)	0.6(0.3-1.1)	208(75.1)	0.7(0.5-1.1)	0.096
Distress from at least one psychological symptom	241(63.9)	75(48.4)	**0.5(0.4-0.8)**	341(87.2)	**4.2(2.8-6.3)**	90(100)	Notestim.	<.001
Feeling sad	143(38.0)	42(25.1)	0.7(0.4-1.0)	247(58.9)	**2.6(1.9-3.6)**	57(67.1)	**3.4(2.0-5.8)**	<.001
Worrying	162(41.9)	50(29.9)	0.7(0.4-1.0)	164(41.7)	1.0(0.7-1.3)	54(63.5)	**2.2(1.3-3.7)**	<.001
Feeling irritable	87(25.9)	11(8.7)	**0.3(0.1-0.6)**	145(42.0)	**2.1(1.5-3.0)**	18(54.5)	**3.5(1.6-7.5)**	<.001
Feeling nervous	116(33.0)	16(11.8)	**0.3(0.2-0.5)**	146(42.6)	**1.6(1.2-2.3)**	22(46.8)	1.7(0.9-3.3)	<.001
**Died at the place of wish** ^**d**^	**221(75.4)**	**214(87.3)**	**1.8(1.1-2.9)**	**176(65.9)**	**0.6(0.4-0.8)**	**67(78.8)**	**1.2(0.6-2.1)**	**<.001**

Missing data:

Physical symptoms were measured on five levels in the original questionnaire (not at all, a little bit, somewhat, quite a bit or very much), variables were later recoded into two categories: quite a bit and very much vs all others; Missing values for physical symptoms Distress from at least one physical symptom: n < 29%; Physical symptoms: lack of appetite: n < 28%; lack of energy: n < 26%; pain: n < 17%; drowsy: n < 19%; constipation: n < 21%; dry mouth: n < 22%; difficulty breathing: n < 19%.

Psychological symptoms were measured on four levels in the original questionnaire (rarely, occasionally, frequently or almost constantly), variables were later recoded into two categories: frequently and almost constantlyvs all others; Distress from at least one psychosocial symptom: n < 51%; Psychosocial symptoms: feeling sad: n < 49%, worry: n < 50%, irritable: n < 59%, nervous: n < 57%.

Died at the preferred place of wish: information available for 44% of the patients.

^a^χ^2^test on cross-country differences.

^b^From multivariate logistic regression models. For these analyses, we compared end-of-life care between patients with cancer in the four countries, with Belgium as the reference category, and adjusted differences in place of death, age, gender, types of malignancy and the number of GPs contact in the last week of life. Odds ratios in bold are statistically significant at p < 0.05.

^c^For at least one symptom.

^d^If the wish of the patient was known to the GP. For the death at the place of wish, we adjusted differences in the longest place of residence, number of GPs contact in the last week of life, age, sex and types of malignancy.

Results from multivariate analyses confirmed cross-country differences on symptoms including pain (more in IT [2.0] & ES [3.2]), dry mouth (more in IT [1.6] & ES [2.3]) and, feeling sad (more in IT [2.6] & ES [3.4]). Patients in NL were more likely (OR 1.8) and patients in IT (0.6) less likely to die in their preferred location compared with BE.

## Discussion

Overall In all countries, four-fifths of cancer patients lived at home or with family in their last year of life. However, the study shows that transitions between care settings at the end of life are common in all countries i.e. more than half over the last three months of life and between 17-27% in the last week of life, and one third of patients (except in NL) died in hospital. There was also a substantial amount of cross-country variation in the provision of end-of-life care to cancer patients even though 80-95% had palliative care as an important treatment goal in their last week of life. While GPs were more strongly involved in palliative care in NL than in other countries, specialist palliative care services were used less often. End-of-life topics were less often discussed and preference for place of death was less often known by the GPs in IT and ES compared with BE and NL. More than 88% of all patients in all countries were estimated to have distress from at least one physical symptom in the final week of life and more than half of cancer patients from at least one psychological symptom.

### Strengths and weaknesses

Strengths of the study include the administration of an analogous research design across countries and the weekly registration keeping recall bias limited, resulting in a robust four-country database of deaths, comparing actual end-of-life care practices. This information supplements the existing data from death certificates or cancer registries [27], hence can serve as an important basis for organisational planning. However, some limitations should be noted. Selecting GPs as the source of information implies underestimation of certain types of care is possible. Nursing homes were excluded in the Netherlands, therefore elderly cancer patients might be underrepresented, although Dutch nursing homes are mainly occupied by people with neurodegenerative disorders [28]. Variations in medical practices exist across countries [29] and the quality of specialist palliative care services was not measured. Also, the questionnaire was kept short and further details on care provision were not available. Even though GPs could offer a macro view of the end-of-life care received by their patients, caregiver-reported outcomes might be more accurate for some items such as caregiver burden and patients’ psychological symptoms in the last week of life. Currently these items were based on ‘GPs’ perception’ after death, and therefore should be interpreted with caution as GPs might under/over-estimate the burden of care.

### Common challenges in end-of-life care

One important common challenge concerns transitions between care settings, which were common during both the final three months (more than half in all countries) and the last week (between one in six and one in four cases) of life. A considerable number of patients (from a third to half) continued to be admitted to hospital in the last week of life and eventually died there. One third or more of informal caregivers of cancer patients were perceived as being overburdened (between 31% and 79%).

Transitions between care settings and terminal hospital admissions are incongruent with the wishes of most patients to die in familiar surroundings and may not only adversely affect the quality of care and the quality of dying of the patient [30–33] but also influence the quality of life of informal caregivers [34, 35]. Although it is unclear from our study why these transitions took place, the results do show that all countries, though the Netherlands least, are struggling to meet most cancer patients’ preferences for dying at home, often due to late hospital admissions. While most palliative care policies of EU countries advocate avoiding hospital death, these results call for the need to understand how this goal can be attained.

### Cross-country differences in palliative care provision and end-of-life communication

Palliative care is organised differently in each of the four countries [7] and our results demonstrate large variations in the care delivered in the final three months. The Netherlands showed a lower percentage of specialist palliative care provision (one third vs. half or more in the other countries) and a stronger GP role in providing end-of-life care for cancer patients. In other countries, the role of specialist palliative care services in counselling regular caregivers is more pronounced in the final months of life. The emphasis on GPs being the primary palliative care providers in the Netherlands [36, 37] –noticeably in education and policy – might be a possible explanation for this difference. Existing research showed that in some cases the involvement of specialist palliative care [38] might increase the proportions of home death, but this cannot be verified by our existing data. These differences might also be related to cultural, legal, societal and organisational variations in care [39–41]. Future studies need to shed light on the interplay between these factors to explain the variations we found.

GPs in the four countries engaged in conversations with their patients concerning prognosis, spiritual issues, palliative care and other end-of-life care issues to various degrees. This illustrates the huge variations in the topics discussed at the end of life in Europe corresponding with results found in previous studies in several other populations [17, 42]. While in all countries physical complaints were frequently discussed, in some such as Belgium and the Netherlands incurability of illness and life expectation were also often discussed, which was not the case in Italy or Spain. Though standardisation in communication might not be feasible due to factors like cultural differences, it would be enlightening to find out how GPs in Belgium and the Netherlands approached patients in these difficult conversations [4], for example whether communication guides could increase physician/patient communication [43, 44].

Furthermore, while all the four countries we studied affirm palliative care as a right for all patients, due to the differences in existing healthcare systems the content of care differs across the countries. Though the present study could not provide answers about the quality of care received by patients in these countries e.g. satisfaction of family, cost and benefit analyses, differences found in outcomes such as place of death and the number of contacts with patients in the last week of life might reflect the specifics of palliative care organisation, such as strong primary care in the Netherlands and the more frequent use of specialist palliative care in Belgium.

### Circumstances of dying

Although the results concerning symptoms in the last week of life should be interpreted with caution considering that they were rated by GPs rated the symptoms in all countries GPs indicated that there is a high prevalence of symptom distress in the last week of life. This might reflect a common problem of symptom control in all countries. On the other hand, the proportions of missing values for physical and psychological symptoms were higher than other items in the questionnaire, which might reflect the limitation of using GPs in reporting them. Symptoms and distress levels reported by patients themselves are more accurate than those rated by proxies, including GPs, nurses or families. The present study did not include patients reported symptom burden (reporting could be burdensome at the end of life) and thus the results canonly be interpreted as what had been perceived by the caring GPs in the final months of life.

### Implications for practice, policy and future research

The latest EAPC Atlas for Palliative Care [7] brings encouraging news to by illustrating recent changes such as the development of postgraduate courses in Belgian universities, the updated Dutch palliative care guidelines, the growth of palliative care support teams in Italy and a Spanish law (applicable in three regions) affirming citizens’ rights to palliative care. Nevertheless, our results revealed that hospitalisations and transitions remain frequent. A lot of cancer patients also had a number of burdensome symptoms at the end of life according to their GPs, which suggests the need for the support of clinicians in assessing the distress of patients. Also, several country-differences became apparent such as in communications and types of palliative care delivery. National palliative care organisations may wish to consider adjusted policies or guidelines for GPs to improve their skills in end-of-life communications and countries could benefit from learning from each other to improve care.

## Conclusion

Although palliative care was the main treatment goal for most cancer patients at the end of life in all four countries, frequent late hospital admissions and the symptom burden experienced in the last week of life indicates that further integration of palliative care into oncology care is required in many countries.

## Consent

Written informed consent was obtained from the patient for the publication of this report and any accompanying images.

## Abbreviations

GP: General practitioners
ORs: Odd ratios
EOL: End-of-life
WHO: World Health Organisation
BE: Belgium
NL: the Netherlands
IT: Italy
ES: Spain.
